# Supramolecular Deconstruction of Bamboo Holocellulose via Hydrothermal Treatment for Highly Efficient Enzymatic Conversion at Low Enzyme Dosage

**DOI:** 10.3390/ijms231911829

**Published:** 2022-10-05

**Authors:** Xinyan Wang, Peng Wang, Yan Su, Qiyao Wang, Zhe Ling, Qiang Yong

**Affiliations:** 1 Innovation Center of Efficient Processing and Utilization of Forest Resources, College of Chemical Engineering, Nanjing Forestry University, Nanjing 210037, China; 2 State Key Laboratory of Pulp Paper Engineering, South China University of Technology, Guangzhou 510640, China

**Keywords:** hydrothermal treatment, bamboo holocellulose, supramolecular structures, enzymatic conversion

## Abstract

Hydrothermal pretreatment (HTP) has long been considered as an efficient and green treatment process on lignocellulosic biomass for bioconversion. However, the variations of cellulose supramolecular structures during HTP as well as their effects on subsequent enzymatic conversion are less understood. In this work, bamboo holocellulose with well-connected cellulose and hemicelluloses polysaccharides were hydrothermally treated under various temperatures. Chemical, morphological, and crystal structural determinations were performed systematically by a series of advanced characterizations. Xylan was degraded to xylooligosaccharides in the hydrolyzates accompanied by the reduced degree of polymerization for cellulose. Cellulose crystallites were found to swell anisotropically, despite the limited decrystallization by HTP. Hydrogen bond linkages between cellulose molecular chains were weakened due to above chemical and crystal variations, which therefore swelled, loosened, and separated the condensed cellulose microfibrils. Samples after HTP present notably increased surface area, favoring the adsorption and subsequent hydrolysis by cellulase enzymes. A satisfying enzymatic conversion yield (>85%) at rather low cellulase enzyme dosage (10 FPU/g glucan) was obtained, which would indicate new understandings on the green and efficient bioconversion process on lignocellulosic biomass.

## 1. Introduction

As the most abundant renewable resource in nature, lignocellulosic biomass has received considerable interest among researchers. To achieve a full application of lignocellulose, its facile deconstruction and degradation of the major components have been proved to be necessary by a bunch of physical, thermo-chemical, or biological treatments [1,2,3]. The bioconversion of its main component, cellulose, to fermentable glucose units is one of the promising goals for overcoming energy shortages and environmental problems [4,5].

Cellulose is a polymer composed of linear β-(1→4) glucosidic linkages, exhibiting rather high resistance to enzymatic or chemical degradation [6]. It is majorly due to the oriented arrangements of glucan chains connected tightly by inter- and intramolecular hydrogen bonds, forming a unique two-phase structure with crystalline regions and amorphous parts [7,8]. Meanwhile, the elementary fibrils consisting of glucan chains further aggregate into larger microfibril bundles in plant cell walls [9,10]. The aggregated structures are often embedded in amorphous hemicelluloses matrixes via van der Waals and hydrogen bonding linkages, which regulate the fibril formation and offer high mechanical properties of cellulose materials [11]. However, the natural aggregations of the polysaccharides together with the highly crystalline domains also bring in difficulties for enzymatic bioconversion, which is commonly described as one of the main causes of lignocellulose biomass recalcitrance [12,13,14].

Attempts have been put forward to overcome biomass recalcitrance and to fulfill the compositional fractionation [15,16]. Among them, HTP is considered as the most economical, feasible, and eco-friendly method, which has been researched for decades [17,18,19]. During HTP, pure water that serves as a solvent is usually mixed with biomass and heated to 130–230 °C for various time periods [20]. At high temperature, water releases H^+^, acting as a weak acid for treatments on lignocellulose [21]. Hemicellulose polysaccharides are the most susceptible to HTP, which causes the cleavage of glycosidic linkages and forms fractions with varied molecular weights [22,23]. Limited impacts were found for HTP on lignin except for a minor proportion of depolymerization [24]. The removal of the amorphous matrix may increase the relative crystallinity index (CrI) of the treated samples. Concerning highly crystalline and aggregated cellulose microfibrils, HTP was proved to efficiently expand the cellulose crystallite sizes and increase the lateral dimensions [18,25]. This phenomenon is accompanied by a decreasing degree of polymerization (DP) and opening of pores for a higher specific surface area, which is favorable for bioconversion. By removing the surrounding non-cellulosic matrix, cellulose macromolecules tend to co-crystallize into twice the initial crystallite size with increasing HTP temperature [26]. The co-crystallization was further proved to happen on crystalline cellulose rather than amorphous parts. In the presence of excessive water with a treatment temperature above 180 °C, the co-crystallization of monoclinic crystals from native cellulose I led to the transformation into a pseudo-orthorhombic form [27]. More recently, decrystallization or pyrolysis of crystalline cellulose was found to take place until the HTP temperature reached >220 °C, much higher than the degradation temperature of amorphous cellulose, suggesting its higher resistance to HTP [28,29]. The destruction of cellulose crystallinity was observed at a high HTP temperature of 190 °C as well as the formation of some cellulose nanocrystals and furfural, which confirmed the supramolecular structural variations of cellulose after HTP [30,31]. Thus, the high obstacles to the bioconversion process resulting from crystalline cellulose are essential to be determined and solved. Moreover, the simultaneous fractionation of the hemicellulose matrix from aggregated elementary/micro cellulose fibrils and its inner connection mechanisms with crystal cellulose chains needs to be further understood.

Herein, moso bamboo with condensed cellulose fibrils aggregation and high crystallinity was used as raw material for HTP under different temperatures [32]. The supramolecular structural variations of crystalline cellulose along with the multi-scale separation of cellulose aggregations were evaluated by a series of analytical methods. The present article also explored the chemical and molecular structures of the degraded hemicelluloses during HTP, which may explain the supramolecular structural changes on moso bamboo polysaccharides. In addition, the proposed interpretation for the coupling deconstruction behavior would provide a deeper understanding of cellulose and hemicellulose matrix interactions in plant cell walls as well as their contributions to biomass recalcitrance.

## 2. Results and Discussion

### 2.1. Chemical Compositional Changes of Bamboo Holocellulose

HTP is known as an efficient pretreatment process to extract hemicelluloses polysaccharides from biomass for enhanced bioconversion. Chemical compositions of bamboo holocellulose as well as samples treated at 120 °C (C1), 150 °C (C2), and 180 °C (C3) were elucidated in Table 1. It is seen that the main hemicellulose content in moso bamboo is xylan, occupying nearly 20% of the raw material. The value was visibly reduced with the increasing treatment temperature, proving that hemicelluloses are quite susceptible to hot water extraction. The xylan removal of C3 reached 83.6%, corresponding to the resulting content of xylan remaining in the solid residue after the HTP. Meanwhile, the relative proportion of the cellulose component rose from an initial 60.3% (HC) to 83.5% for C3, as well as maintaining a high glucan recovery yield (94.6%). The chemical variations of the solid samples were also revealed by a Fourier transform infrared spectrometer equipped with an attenuated total reflection accessory (ATR-FTIR) (Figure S1). Typical peaks for carbohydrates were visible at 3400 cm^−1^ and 1031 cm^−1^, respectively, corresponding to C–O stretching and O–H bending in polysaccharides [33]. The effects of HTP on the hemicellulose were located at the peaks of 1736 cm^−1^ and 1637 cm^−1^, which greatly decreased with an increasing treatment temperature and nearly disappeared for C3 [34]. The peaks were assigned to the stretching vibration in acetyl groups and the OH bending vibration of hydration water in xylan-type polysaccharides [35]. This phenomenon strongly supported the results of xylan removal from the compositional analysis. Regarding the cellulose component, HTP had no effect on the Iβ allomorph due to the peaks at 712 cm^−1^. However, the slightly reduced peak at the 2900 cm^−1^ C–H stretching vibrations represented the effects on the intra- or intermolecular bond of cellulose after HTP. The peaks at 1430 cm^−1^ showed sensitivity to the crystalline part of cellulose and showed a slight decrease for C2 and C3, indicating the disturbing of cellulose crystals in the hydrothermal environment [15].

DP is a key factor affecting the properties and applications of cellulose materials [36]. DP was tested and calculated via cupriethylenediamine (CED) dissolution and viscosity method as reported previously [37]. Xylan and glucan content in holocellulose based on the compositional analysis were considered in the calculation of DP values. Lower DP refers to a less complicated network of fibers and weaker hydrogen bonding of cellulose, promoting its susceptibility to hydrolysis [38]. In this work, hemicellulose polysaccharides were included in the calculation of DP, which turned out to be 4420 for raw HC. The value is slightly higher than the α-cellulose or cellulose fibers extracted from bamboo, simply due to the interactions of xylan with the glucan chains. The degradation of xylan and increased relative amount of cellulose had no obvious effect on the samples after HTP at 120 °C and 150 °C. However, the DP value for C3 showed a great drop to 3858, suggesting the weakening of intermolecular hydrogen bonding and the breaking of cellulose chains at 180 °C. It agrees with the above chemical compositional analysis and further studies deserve to be performed on the structural changes of cellulose microfibrils and the extracted xylan.

### 2.2. Morphology and Microfibrillar Deconstruction

**Figure 1:**
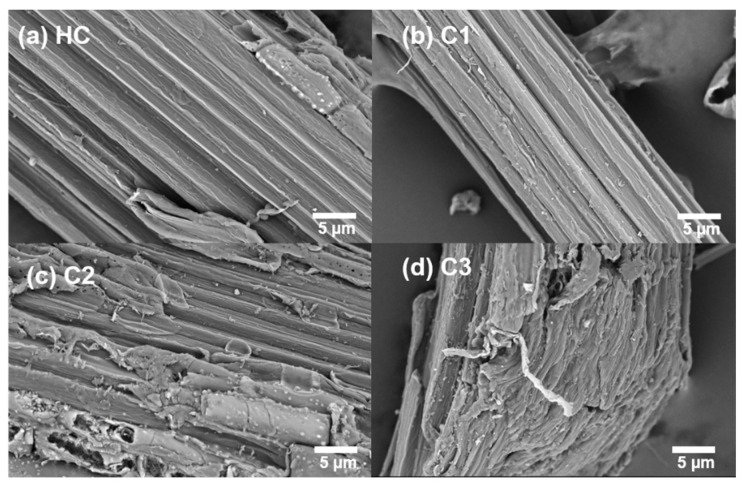
SEM images of the sample HC (**a**), C1 (**b**), C2 (**c**) and C3 (**d**) before and after HTP (Scale bar = 5 μm).

Holocellulose was observed by SEM to determine the morphological changes after HTP (Figure 1). Raw HC exhibited a flat and smooth surface of the fibers, which were arranged orderly. The cellulose microfibrils aggregated tightly by the linkages with the hemicellulose matrix by both hydrogen bonding and van der Waals forces. The regular stacking of cellulose fibrils embedded in the matrix formed a high resistance to chemical or biological treatments. The cellulose fiber bundles became loosened after HTP, especially under a treatment temperature higher than 150 °C. A rough surface appeared, along with the cracks and degraded hemicellulose fractions. With the increasing temperatures, C3, which was treated at 180 °C, showed not only a rougher surface, but also the breaking of fiber bundles, generating larger pores. Swelling of the fibers was more significant for this sample, causing more irregular connections between the microfibrils. The destroyed structure of cellulose aggregates would be beneficial for the adsorption and degradation of cellulase enzymes followed by HTP.

**Figure 2:**
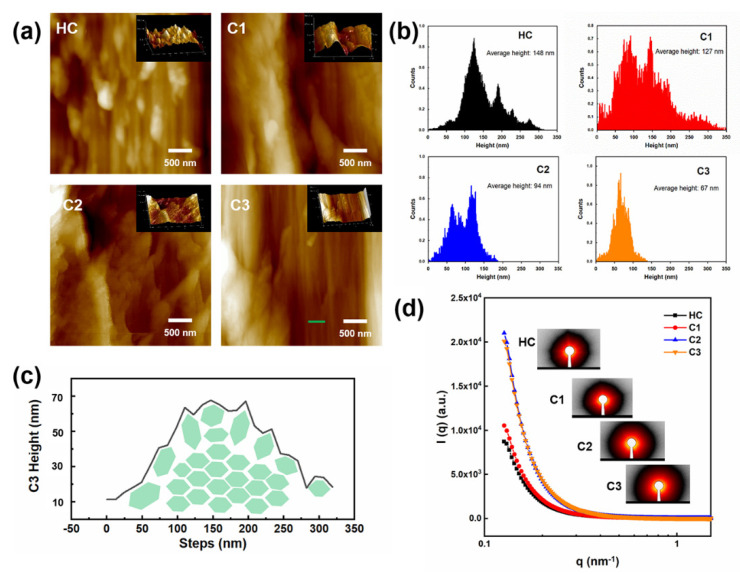
AFM images (**a**) and the average height calculations (**b**) of the holocellulose slices before and after HTP; schematic model of microfibrils based on step scanning from C3 AFM image (**c**); SAX patterns and corresponding 2D images (**d**).

To explore the inner mechanisms of microfibrillar aggregations and their deconstruction after HTP, AFM images and small-angle X-ray scattering (SAXS) patterns are provided in Figure 2. Observations on holocellulose slices in the longitudinal direction are presented by phase images of AFM, which may reveal the microfibrillation behavior of the samples. The xylan matrix laying on the surface of cellulose microfibrils is visible for HC. Three-dimensional images illustrate that HC had a coarser structure with more complex distributions of the components. HTP, with increasing temperatures, would contribute high amounts of xylan removal, which exposed more fibrillary cellulose. The larger area of cracks and thinner microfibrils were present, especially for the C3 sample. By counting and plotting the height of the AFM images, the average diameters of microfibril bundles can be calculated, which can reflect the effects of HTP on the cellulose aggregates separation [39]. It is seen that HTP significantly contributed to thinner microfibrillar aggregates as well as a narrower diameter distribution. The phenomenon appeared accompanied by the high amount of xylan removal. The average height of C3 slices was reduced to 67 nm, which means the co-aggregation of 10–20 cellulose elementary fibrils (3–5 nm in diameter) [40]. The step count of C3 was achieved by analyzing the green line area in Figure 2a. A proposed hexagon model of microfibrils was drawn based on the height pattern [41]. Swelling of the surface cellulose was hypothesized as well as some rotation of microfibrillar orientations. This may generate more pores and gaps between the cellulose microfibrils, which was proved by the higher scattering intensity in SAXS patterns and the 2D images. The slight bump of HC at around q = 0.13 nm^−1^ disappeared for samples by HTP, which probably can be attributed to the gaps or pores with diameters larger than the scattering limit [42].

### 2.3. Cellulose Crystal Structures 

**Figure 3:**
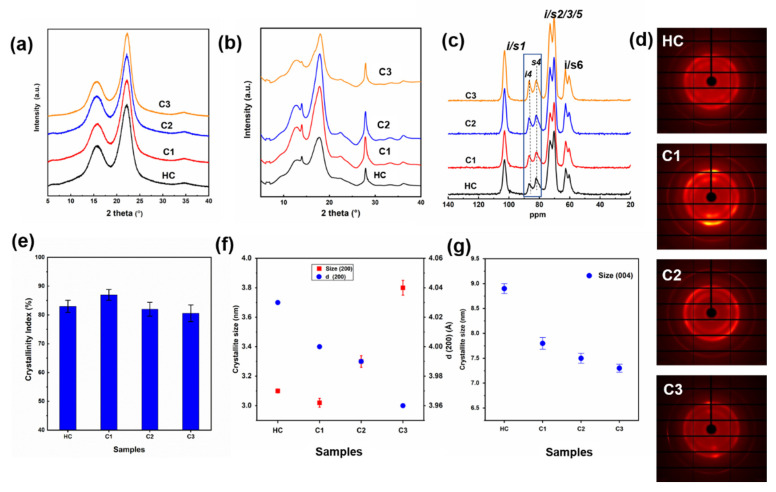
One-dimensional XRD (**a**)/WAXS (**b**) patterns, ^13^C NMR spectra (**c**), and the two-dimensional (**d**) images of the holocellulose before and after HTP (C1, C2 and C3); calculated CrI (**e**), crystallite sizes, and d-spacings (**f**,**g**) perpendicular to different lattice planes.

With the deconstruction of the microfibrillar aggregations, inner crystal variations of cellulose deserved deeper determination. Laboratory X-ray diffraction (XRD) and synchrotron wide-angle X-ray scattering (WAXS) were both performed on the bamboo holocellulose powders. The two patterns exhibited similar peak shapes of cellulose Iβ. The result agrees with the peak at around 104 ppm in nuclear magnetic resonance (NMR) spectra, which corresponds to the Carbon-1 region of highly crystalline cellulose Iβ (Figure 3c) [43]. Additionally, WAXS patterns provided sharper peaks, especially at around 2θ = 28° representing (004) a lattice plane perpendicular to the direction of glucan chains. The 2D images in Figure 3d displayed more information on cellulose crystal structural changes. There appeared to be an increase of average brightness for the C1 sample, indicative of more crystals being generated after HTP at 120 °C. Meanwhile, the preferred orientation of the crystals was proved by the symmetric bright dots in the vertical direction. A similar phenomenon was discussed in ball-milled cellulose with a short milling time [44]. The pattern that resembles the raw bamboo fiber bundles may have resulted from the partial xylan removal [45], which caused the exposure of the assembling cellulose crystals and inner glucan chains. The exposure of inner cellulose chains can also be proved by the increased NMR peak at 88 ppm due to the inner cores of the cellulose Carbon-4 region. It was reported that more crystalline regions are located in inner cores of cellulose microfibrils [46]. Thus, HTP could cause the increasing CrI as calculated from NMR spectra based on the peaks’ deconvolution and Equation (2) (Figure S2), which correspond to previous studies [22,47]. 

When focusing on the darker 2D patterns and more isotropic crystal arrangements, however, this phenomenon may be due to slightly decreased CrI, and was proved by the quantitative calculations (Figure 3e). Removal of amorphous hemicelluloses increased the CrI of C1 to 87%, followed by a return to ~81% for C2 and C3, similar to the value of HC. No matter the raw materials used in previous work, the delignification and the removal of the hemicellulose matrix during HTP played major roles in promoting the CrI. However, it should be noted that the hemicellulose removal also favored the breaking of hydrogen bonding between hemicellulose and cellulose, causing interfacial crystalline disorder [48]. The decrystallization behavior was supported by the slightly reduced CrI of C2 and C3. However, the decrystallization is limited, referring to other crystalline changes in the inner core region of cellulose.

Crystal structural changes regarding the crystallite sizes and d-spacings of different lattice planes based on the Rietveld analysis of XRD patterns were illustrated in Figure 3f,g. The crystallite sizes of the (200) lattice plane that reflects the main peak in the XRD patterns showed a great increase from 3.1 nm to 3.8 nm of C3. The possible mechanism was reported to be the co-crystallization of the adjacent crystallites, along the bonds at crystallite interfaces [48]. It may be motivated by the removal of the interfacial hemicellulose. The migration and intercalation of water molecules at high temperatures that caused the crystallite fusion were also a possible reason for the larger crystallite sizes after HTP [49,50]. Additionally, the swelling was more prominent in the presence of excess water. The expansion of lateral crystallite sizes of the (200) lattice plane directly led to a slight shrinkage of d-spacings, which reflects the distance between the adjacent (200) planes. It could be explained by the swelling of the crystallites, thus occupying the space between the glucan chains. There was an exception for the C2 sample which had both a decreased crystallite size and d-spacings perpendicular to the (200) plane. The difference was probably due to the limited xylan removal, thus resulting in a less predominant expansion of cellulose crystals.

Interestingly, the decrease of crystallite sizes along the axial direction ((004) lattice plane) was found by calculating WAXS peak width based on the Scherrer equation (Equation (4)). A similar phenomenon was reported in our previous study on levulinic acid-based deep eutectic solvents (DES) pretreatment on moso bamboo (MB) [51]. This could be the major reason contributing to more fractions of amorphous cellulose and reduced CrI for samples after HTP, together with the xylan removal. The lowered crystal sizes along the axial direction may also cause the generation of more pores and cracks of the microfibrils of a larger size, corresponding to the above SAXS patterns. Anisotropic variations of crystallite sizes in diverse directions indicated the occurrence of broken cellulose aggregates during the HTP, which would facilitate the following enzymatic conversion of cellulose.

### 2.4. The Effects of HTP on Xylan Extraction

HTP has been generally discussed concerning the capabilities of extracting hemicellulose and degrading it into oligosaccharides [52]. The molecular weight (Mw) of an oligosaccharide sample can be determined by GPC. It is seen in Table 2 that Mw of extracted xylan or xylooligosaccharides (XOS) and their polydispersity index (PDI) decreased visibly with the increasing treatment temperature. HTP under 180 °C resulted in the highest ratio of XOS (DP = 2–6) as calculated from Mw. Meanwhile, the increase of retention time from GPC indicated the smaller Mw and fewer components in the hydrolyzates of CX3, which had a narrower Mw distribution (Figure S3). The extracted xylan with different Mw from hydrolyzates was further dispersed and dissolved in the deionized water to analyze their structures. Xylan with high Mw and PDI (CX1) showed as fewer homogeneous particles compared to CX3, which was mainly composed of XOS with lower PDI, although the particles’ sizes seemed to be slightly larger than CX1 as determined by both transmission electron microscopy (TEM) and dynamic light scattering (DLS). The morphological characterizations were similar to previously reported images of xylan extracted from steam and xylanase-treated corncob [53].

**Figure 4:**
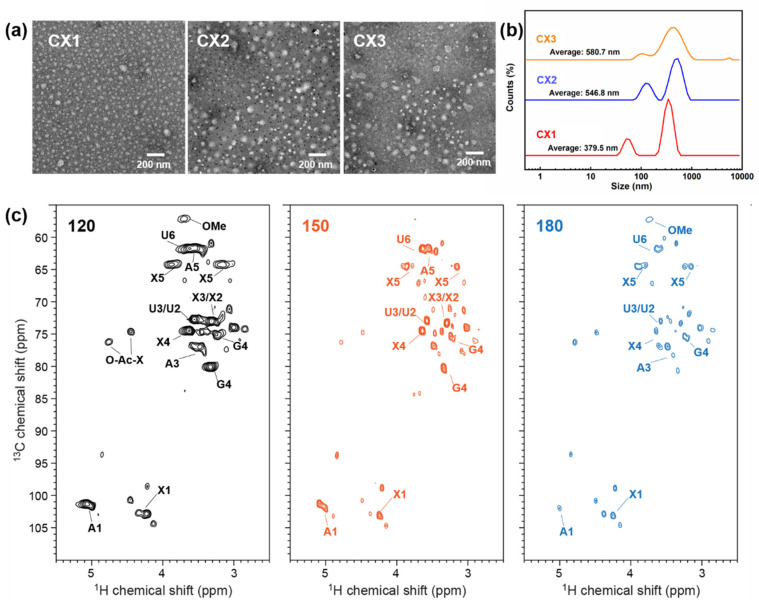
TEM images (**a**) and DLS results (**b**) of the hydrolyzates after HTP; 2D HSQC NMR spectra of hemicelluloses fractions (**c**).

The structure of XOS samples was verified by 2D-HSQC spectra. As shown in Figure 4c, there appeared to be several signals for the hydrolyzates after HTP, including β-(1→4)-D-xylopyranoside (X) of internal xylan, β-(1→4)-D-xylopyranoside with reducing end, β-(1→4)-D-xylopyranoside with non-reducing end, 2-O-acetyl-β-D-xylopyranoside (O-Ac-X), 3-O-acetyl-β-D-xylopyranoside, (1→4)-α-D-xylopyranoside, (1→4)-β-D-xylopyranoside, α-(1→4)-L-arabino-furanoside (A), 4-O-methyl-a-D-Glcuronic acid (U) as well as some degraded low-Mw cellulose fractions [54,55,56]. The clear degradation of hemicelluloses fractions in the spectra suggests that XOS was mainly derived from 4-O-methylglucuronoarabinoxylan. Notably, the correlation signals for Xylose, xylobiose, xylotriose, xylotetraose, and xylopentaose as well as 4-O-methyl-a-D- Glcuronic acid declined with the rise of HTP temperature, which is in accordance with the results of larger-extent degradation and hydrolysis of xylan due to the increase of pretreatment intensity [23].

### 2.5. Enzymatic Conversion and Accessibility

**Figure 5:**
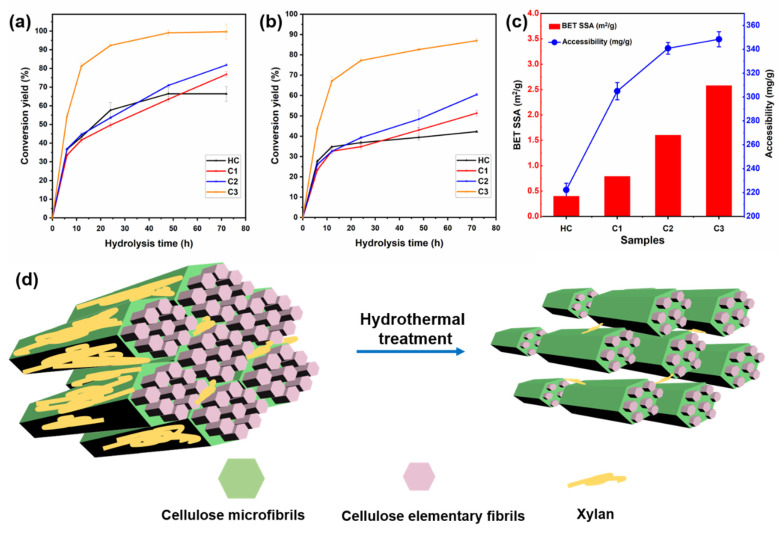
Enzymatic conversion yield of the bamboo holocellulose at the dosage of 20 FPU/g glucan (**a**) and 10 FPU/g glucan (**b**); BET surface area and accessibility of the samples (**c**); and the schematic model of the process of HTP on the bamboo cellulose aggregations (**d**).

The bioconversion performance of the HC samples before and after HTP was evaluated by glucose yields under cellulase enzymatic hydrolysis. As seen in Figure 5a,b, we used two groups of enzyme dosages, 20 FPU/g (filtrate paper activity unit per gram) glucan and 10 FPU/g glucan, to determine the enzymatic conversion of the samples. It turns out that, under different incubation circumstances, there appeared the similar increasing trend of glucose yield for hydrothermally treated HC. After 72 h of hydrolysis, C3 presents a high enzymatic conversion yield of 98.6% at the dosage of 20 FPU/g glucan, indicating a satisfying HTP effect on HC under 180 °C. It should be noticed that, with the low enzyme dosages, C3 still showed 87.1% conversion yield to glucose, which was even higher than the results of thermal-chemical pretreated bamboo samples. Although HC was firstly delignified before HTP in this work, the high conversion yield proved the effective deconstruction of the cellulose aggregation complex. The increased crystallite size perpendicular to the hydrophobic (200) lattice plane favored the cellulase enzymes’ adsorption and subsequent degradation of cellulose chains [57]. Moreover, in the axial direction parallel to cellulose chains, the pores and cracks may provide more space and a larger surface area for the attack of enzymes.

The accessibility of HC samples was determined by both the N_2_ adsorption using Brunauer–Emmett–Teller (BET) surface area analysis and the Congo red dyes staining assay (Figure 5c). It was seen that specific surface area (SSA) detected by the BET method increased notably from 0.40 m^2^/g (HC) to 2.58 m^2^/g (C3), which agrees with the SSA value for physicochemically separated bamboo elementary fibrils [58]. This is strong evidence of the successful deconstruction of the cellulose aggregates by HTP. It also proved the series of changes in supramolecular structure as determined by WAXS/SAXS. The Congo red dye staining assay supported the conclusion of a higher surface area for samples after HTP at higher temperatures. C3 had a high dye accessibility of 348 mg/g, which was similar to the recently reported value of MB treated by dicarboxylic acid-based DES [59]. Although there was more decrystallization behavior that took place in that work, the significant removal of the surface hemicellulose matrix contributed significantly to the increased SSA as well as the accessibility to cellulase enzymes. Meanwhile, the chemical and morphological structural deconstruction after HTP was also proved by the reduction of thermal stability as presented in Figure S4. There was a higher degradation rate and less char remaining during the thermogravimetric analysis (TGA) test, especially for C2 and C3, which were hydrothermally treated at higher temperatures.

As proposed in the schematic model in Figure 5d, HTP induced multi-scale deconstruction on the bamboo holocellulose. The aggregated and condensed structure embedded in the hemicellulose matrix was disrupted by HTP in its chemical composition, morphology, and crystallite assemblies. After the large amount of xylan removal, more cellulose fibers were exposed, relieving the recalcitrance of the bamboo cell wall. Meanwhile, there appeared to be crystal expansion and elementary fibrils separation after HTP, which could increase the surface area of the microfibrils. The cracks in the axial direction of cellulose further destroyed the aggregated structures, favoring the subsequent enzymatic conversion of bamboo cellulose to glucose.

## 3. Materials and Methods

### 3.1. Materials Preparation

Moso bamboo (*Phyllostachys edulis*, MB) was kindly supplied by the National Forestry Center, Yunnan Province, China. The raw materials were air-dried and milled by a laboratory pulverizer to powders and passed through a 20–80 mesh screen. MB powders were then extracted by toluene/ethanol (2:1, *v*/*v*) for 6 h and oven dried at 60 °C overnight to remove the extractives. To focus on the cellulose and hemicellulose polysaccharides in the samples, lignin was removed by the conventional bleaching method [46,60]. Briefly, the extractive-free samples were treated with sodium chlorite/acetic acid at 75 °C for up to 4 h at the mass/liquid ratio of 1:50. The bamboo holocellulose (lignin-free sample, HC) was washed thoroughly and air-dried for further use. The bamboo sticks were treated by the same process as the powders and cut in a longitudinal direction into slices (thickness = 10 μm) by a Leica SM2010R microtome for microstructural observations. Cellulase (SAE0020) with a filter paper activity of 200 FPU/g was purchased from Sigma-Aldrich (Shanghai, China). All other chemical reagents used were analytical grade and purchased from Macklin Chemical Company (Shanghai, China).

### 3.2. HTP and Components Separation

HTP on bamboo holocellulose powders and slices were performed in a stainless-steel reactor with water at the solid/liquid ratio of 1:10. The reactor was put in the oil bath with a heating rate of 1.5 °C/min at the temperatures of, respectively, 120 °C, 150 °C, and 180 °C for 1 h. Subsequently, the pretreated samples were cooled into a cool-water bath for 20 min and filtrated to separate the solid residues and liquid. Residues treated at different temperatures (respectively marked as 120 °C: C1, 150 °C: C2, and 180 °C: C3) were washed by deionized water and stored at 4 °C for further characterizations. The xylan-rich liquids, also called autohydrolyzate, were, respectively, diluted to 5g/L and marked as CX1, CX2, and CX3. To extract the xylan solids in autohydrolyzate, the liquids were precipitated by triple volumes of ethanol (95%) solution for the following characterizations.

### 3.3. Morphology of Bamboo Powder

Morphologies of the powder samples were observed by a Thermo Fisher VolumeScope 2 scanning electron microscope (SEM) under an accelerating voltage of 10 kV. The machine was equipped with multiple kinds of detectors with scattering electron, backscattered electron, and X-ray detectors and the working distance was set as 4.5 mm. Sample preparations were made by oven-drying and spray coating with Au [61], as in previous reports. Atomic force microscope (AFM, Bruker Dimension Edge SPM, Germany) analysis was used to investigate the microfibrillar sizes of the treated slices. The experiment was operated in intermittent contact mode with a scan frequency of 2 Hz. The images were analyzed by Nanoscope Analysis software (Version 1.40). The extracted xylan particles in the autohydrolyzate were detected by a JEM1400 electron transmission microscope (TEM), operating at an accelerating voltage of 160 kV. Samples were dropped on the carbon grids and stained by phosphotungstic acid (2% *w*/*v*) before the TEM observation. The particles were also analyzed by using a dynamic light scattering (DLS) instrument (Malvern Instruments Ltd., Malvern, UK).

### 3.4. Chemical Compositional Analysis

Chemical compositions of HC and samples by HTP were characterized by the National Renewable Energy Laboratory (NREL) protocol [62]. The degree of polymerization (DP) of the raw and treated holocellulose was determined as it was in previous reports [37,63]. An amount of 0.1 g bleached residue was mixed with 20 mL cupriethylenediamine (CED) by shaking in a dark environment until the solids were dissolved. Then, an Ubbelohde viscometer (0.5–0.6 mm) was used to obtain the dissolved liquid intrinsic viscosity at 20 °C ± 0.3 °C. The DP of bamboo holocellulose residues was calculated based on the intrinsic viscosity in CED solution according to the following equation (Equation (1)):

(1) $$\mathrm{DP}={\left(\frac{1.65\left[\eta \right]-116\left[\mathrm{Xyl}\right]}{\left[\mathrm{Glu}\right]}\right)}^{1.111}$$

where [η] is the intrinsic viscosity of the holocellulose residues in CED solution, and [Xyl] and [Glu] are the xylan and glucan content in the sample based on the compositional analysis, respectively.

The chemical analyses were also carried out by a Nicolet 6700 Fourier transform infrared spectrometer (FTIR, Thermo Fisher Scientific, USA) equipped with an ATR accessory (ATR-FTIR). Solid-state ^13^C NMR spectra were acquired using a Bruker Advance III 400 MHz; 9.4 Tesla, Germany) spectrometer with a 4 mm MAS HCN probe. The CP contact time was 1 ms and the magic-angle spinning frequency was 10 kHz. The crystallinity of the pretreated celluloses could be calculated by comparing the region area of NMR spectra (Equation (2)): [64]

(2) $$\mathrm{CrI}=\frac{A_{80-85\;\mathrm{ppm}}}{A_{80-85\;\mathrm{ppm}}+A_{85-92\;\mathrm{ppm}}}\times 100\%\;$$

Thermal stabilities of the solid samples were also varied after HTP and were characterized by thermogravimetric analysis (TGA)/a derivative thermogravimetric analyzer (DTG, TA instruments Q50, USA). All the spectra were normalized for comparison and discussion.

For autohydrolyzate, the molecular weight (MW) and mass distribution were determined by gel permeation chromatography (GPC) that took place within an HPLC system (Agilent 1200, Palo Alta, CA, USA). The mobile phase of the analysis system was KH_2_PO_4_ (600 mM). Average molecular weights were calculated through external calibration based on the retention time of different molecular weight dextran standards. Chemical bond linkages in the extracted xylan were characterized by 2D-HSQC (Heteronuclear Single Quantum Coherence) NMR technology using a Bruker AVANCE 600 MHz spectrometer. An amount of 40 mg of sample were dissolved into 0.5 mL DMSO-d6 prior to spectral acquisition with a spectral width of 1H and 13C for 3497 and 18,750 Hz, relaxation time of 1H for 1.5 s, sampling time for 2.7 s, collection point for 1024, and scanning times for 128 and 257, respectively.

### 3.5. Supramolecular Characterizations on Cellulose

X-ray diffraction (XRD) analysis was performed in reflection mode by a Rigaku Ultima IV X-ray diffractometer equipped with Cu Kα-radiation (λ = 0.15419 nm) for determination of cellulose-rich residues after the HTP. The examples were tested by using a Rigaku Ultima X-ray diffractometer and then analyzed using the pseudo-Voigt peak shape with the MAUD Rietveld program (Materials Analysis Using Diffraction, version 2.7) [65]. The CrI of the samples was calculated based on the fitted results via the equation below:

(3) $$\mathrm{CrI}=\frac{A_{\mathrm{crystal}}}{A_{\mathrm{crystal}}+A_{\mathrm{amorph}}}\times 100\%\;$$

where A_cryst_ is the area below the calculated crystalline cellulose intensity and A_amorph_ is the area below the calculated amorphous intensity.

The d-spacings were calculated by the Bragg equation [66] and crystallite sizes perpendicular to each lattice plane (L_hkl_) of cellulose allomorphs were calculated by the Scherrer equation (Equation (4)): [67]

(4) $$L_{\mathrm{hkl}}=\frac{0.9\lambda }{B_{\mathrm{hkl}}\cos\theta }$$

where λ is the X-ray wavelength (0.15419 nm), B_hkl_ is the angular FWHM (full width of half maximum) of calculated crystalline peaks, and θ is the Bragg angle.

Synchrotron radiation wide-angle X-ray scattering (WAXS) and small-angle X-ray scattering (SAXS) experiments were conducted at beamline BL16B1 at the Shanghai Synchrotron Radiation Facility (SSRF). The wavelength was 0.124 nm and the sample-to-detector distance was 2051 mm (corrected by CeO_2_ monocrystalline powder). The data acquisition time was 60 s for WAXS and 10 s for SAXS patterns. For SAXS pattern, the *q* range was 0.1–1.1 nm^−1^ (or 60–6 nm in d-spacing). Here, *q* is defined as 4πsin θ/λ, where θ is the Bragg angle. Two-dimensional (2D) scattering images were obtained by analysis with Fit2D software from the European Synchronization Radiation Facility.

### 3.6. Enzymatic Hydrolysis and Solid Accessibility Evaluation

The solid residues of raw and pretreated holocellulose were hydrolyzed in 0.05 M sodium acetate buffer with a pH of 4.8. The solid to liquid ratio was set as 10% (*w*/*v*) and the flask was air-shaken at 48 °C and 150 rpm for 72 h. The enzyme loading was set as 20 FPU/g and 10 FPU/g glucan, respectively, in order to investigate the enzymatic conversion performance at a low enzyme dosage. The glucose yield from enzymatic hydrolysis was calculated based on the release of glucan (HPLC, Agilent 1200, Palo Alta, CA, USA) as a percentage of theoretical glucan available in the substrate at different time intervals. The HPLC system contained an Aminex Bop-Rad HPX-87 column. H_2_SO_4_ (5 mM) was used as the eluent (0.6 mL/min) during analysis. All assays were performed in triplicate.

The specific surface area (SSA) of the holocellulose was measured according to the nitrogen adsorption method described by Brunauer, Emmet, and Teller (BET) [68] on a BET Surface Area Analyzer (ASAP 2020, Micromeritics). Samples were oven-dried at 90 °C for 2 h before the BET test. A Congo red dye staining assay was also used for the evaluation of accessibility of the enzymes to substrates. The solid residues were thoroughly mixed with different concentrations (0, 0.05, 0.1, 0.5, 1, 2, 3, and 4 g/L) of Congo red solution at a 1% (*w*/*v*) concentration of the substrate in a sealed conical flask. The dyeing process lasted for 24 h in a thermostatic shaker at 150 rpm, 50 °C, followed by measuring the supernatants via a UV/Vis spectrophotometer (Ultrospec 2100, Amersham Bioscience) at 498 nm. The amount dye adsorption was calculated from the remaining content of dye in the solution by the Langmuir adsorption isotherm. The equation is as follows: [69]

(5) $$T=\frac{T_{m}\mathrm{AC}}{1+\mathrm{AC}}$$

where the T is the concentration of the adsorbed dye by the substrate (mg/g); T_m_ is the concentration of the adsorbed dye (mg/g); A is the partition coefficient; and C is the free dye content in the supernatant (g/L).

## 4. Conclusions

In summary, bamboo holocellulose with highly aggregated microfibrils and crystalline structure was treated in a hydrothermal environment at various temperatures. Chemical compositional analysis indicated a large amount of xylan removal with increasing HTP temperatures, which was accompanied by the reduced DP of cellulose from 4420 to 3858. Simultaneously, XOS were generated in the hydrolyzates after HTP, and they were mainly derived from the degradation of 4-O-methylglucuronoarabinoxylan. For cellulose, the major component, there was a slight decrease of the CrI for the treated HC (~80%). However, the crystallite sizes changed anisotropically, with the expanding size perpendicular to the hydrophobic (200) lattice plane from 3.1 nm (HC) to 3.8 nm (C3) and the greatly decreased size in the axial direction. The multi factors from HTP resulted in the weakened hydrogen bond linkages between cellulose molecular chains, which therefore loosened and separated the condensed cellulose microfibrils. Thus, the surface area of the treated bamboo holocellulose increased notably to 2.6 m^2^/g together with the improved accessibility. A satisfying enzymatic conversion yield (>85%) at a rather low cellulase enzyme dosage (10 FPU/g glucan) was obtained for the sample by HTP under 180 °C. Herein, the revisited hydrothermal treatment suggested some new understandings of the supramolecular structural variations of lignocellulosic resources, serving for a more efficient and economically beneficial bioconversion process.

## Figures and Tables

**Table 1:** Chemical compositional analyses and DP calculations of HC powders before and after HTP at 120 °C (C1), 150 °C (C2), and 180 °C (C3).

Samples	Glucan (%)	Xylan (%)	Arabinan (%)	Lignin (%)	Recovery Yield (%)	Glucan Recovery (%)	Xylan Removal (%)	DP×10^3^
HC	60.3	19.9	4.3	6.2	-	-	-	4.4
C1	63.4	18.6	1.3	4.4	93.1	97.9	12.9	4.6
C2	73.5	12.9	-	3.4	79.9	97.3	47.9	4.6
C3	83.5	4.8	-	3.2	68.3	94.6	83.6	3.9

**Table 2:** Weight-average (Mw) and number-average (Mn) molecular weight and polydispersity (PDI, Mw/Mn) in the aqueous phase after HTP.

Samples	Mw	Mn	PDI
CX1	16.8 × 10^3^	10.0 × 10^3^	1.67
CX2	10.9 × 10^3^	9.4 × 10^3^	1.16
CX3	482	458	1.05

## Data Availability

All data generated or analyzed during this study are included in this published article and its additional files.

## Supplementary Materials

The following supporting information can be downloaded at: https://www.mdpi.com/article/10.3390/ijms231911829/s1.
