# Supplementary material for: Supramolecular Deconstruction of Bamboo Holocellulose via Hydrothermal Treatment for Highly Efficient Enzymatic Conversion at Low Enzyme Dosage
Source: Int J Mol Sci. 2022 Oct 5;23(19):11829. doi: 10.3390/ijms231911829 (PMC9570373; doi:10.3390/ijms231911829)
Supplement: Supplementary file 1 [file ijms-23-11829-s001.zip › ijms-1949386-supplementary.pdf]

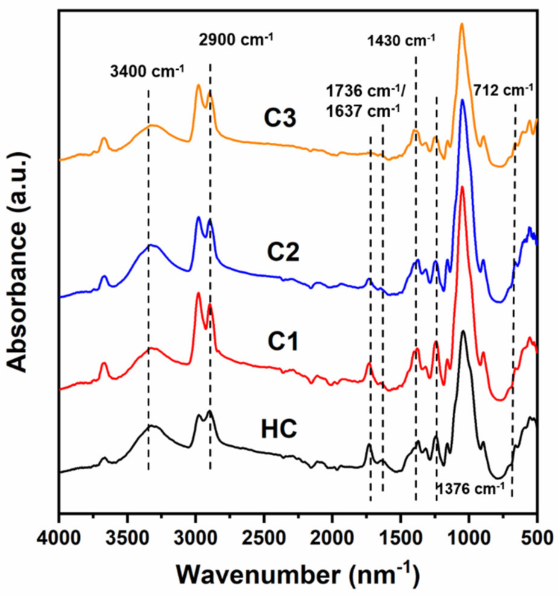


**Figure S1.** ATR-FTIR spectra of the holocellulose samples before and after HTP.

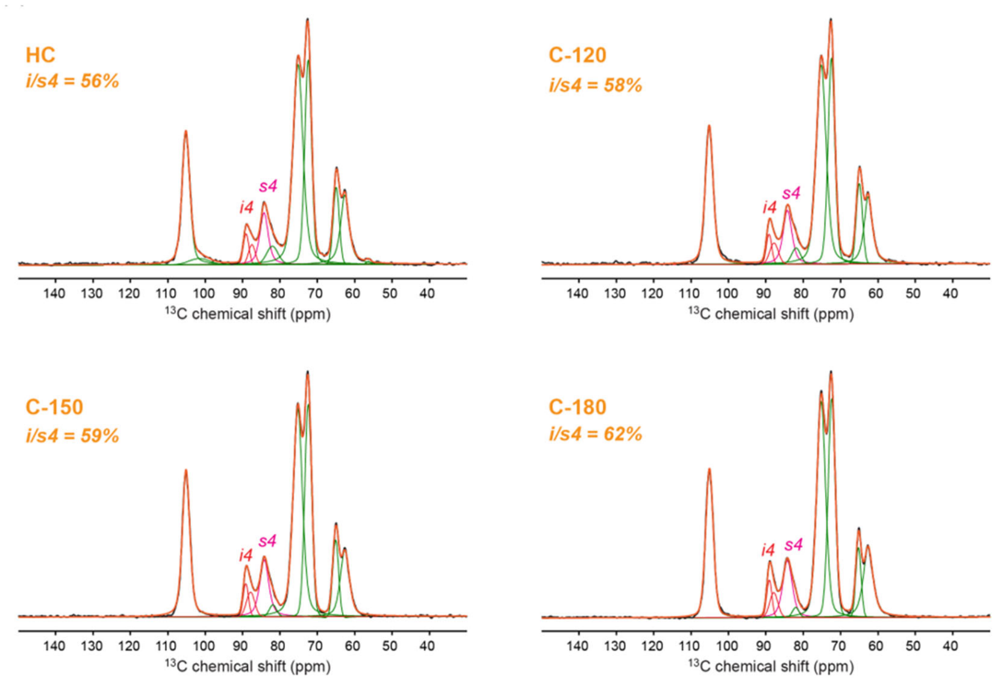


**Figure S2.** Peaks deconvolution of the $^{13}C$ NMR spectra of holocellulose samples before and after HTP**.**

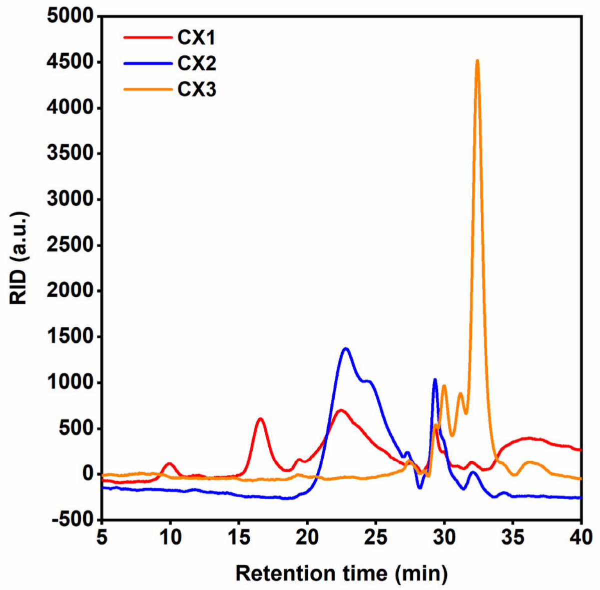


**Figure S3.** Gel Permeation Chromatography (GPC) testing results for the extracted xylan after HTP.

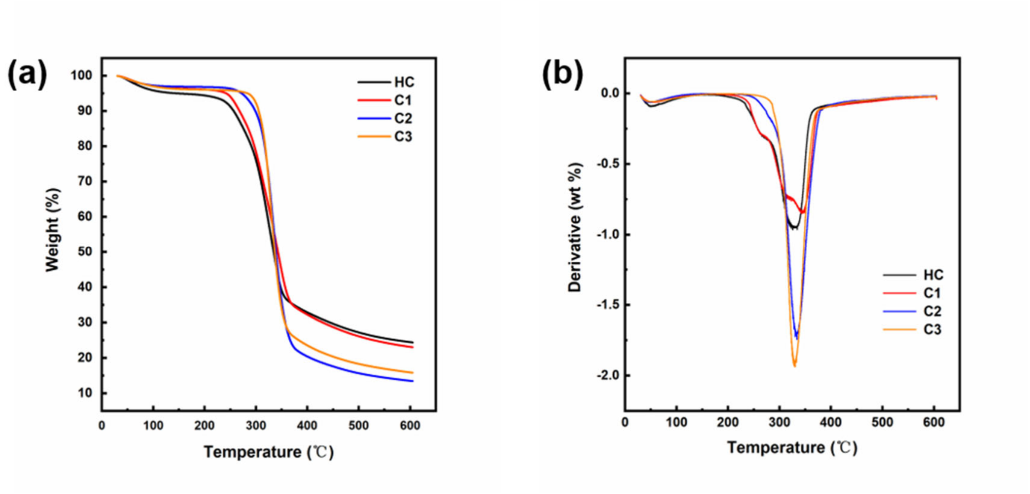


**Figure S4.** TGA (**a**) and DTG (**b**) curves of the holocellulose samples before and after HTP; the temperature increased from 30 ℃ -600 ℃.
